# Experiences of healthcare staff providing community-based mental healthcare as a multidisciplinary community mental health team in Central and Eastern Europe findings from the RECOVER-E project: an observational intervention study

**DOI:** 10.1186/s12888-021-03542-2

**Published:** 2021-10-24

**Authors:** Catharina Roth, Michel Wensing, Martina Rojnic Kuzman, Sarah Bjedov, Sara Medved, Ana Istvanovic, Danijela Stimac Grbic, Ivana Pavic Simetin, Aleksandar Tomcuk, Jovo Dedovic, Tatijana Djurisic, Raluca Ileana Nica, Tiberiu Rotaru, Antoni Novotni, Stojan Bajraktarov, Milos Milutinovic, Vladimir Nakov, Zahari Zarkov, Roumyana Dinolova, Bethany Hipple Walters, Laura Shields-Zeeman, Ionela Petrea

**Affiliations:** 1 Department of General Practice and Health Services Research, Heidelberg University Hospital, Marsilius Arcades, West Tower, Im Neuenheimer Feld 130, 69120 Heidelberg, Germany; 2 Clinic for Psychiatry and Psychological Medicine, Zagreb University Hospital Centre, Kišpatićeva ul. 12, 10000 Zagreb, Croatia; 3 Zagreb School of Medicine, University of Zagreb, Zagreb, Croatia; 4 Croatian Institute of Public Health, Rockefellerova ul. 7, 10000 Zagreb, Croatia; 5 Health Institution Special Psychiatric Hospital Dobrota Kotor, Dobrota bb, 85330 Kotor, Montenegro; 6 Public Health Institute of Montenegro, Dzona Dzeksona bb, 81000 Podgorica, Montenegro; 7 Institute Liga Romana pentru Sanatate Mintala, Sos. Mihai Bravu 90-96, Bucuresti-Sector 2, Romania; 8 Siret Psychiatric Hospital, Strada 9 Mai 5, 725500 Siret, Romania; 9 University Clinic of Psychiatry, Мајка Тереза 17, Mother Teresa 17, Skopje, 1000 North Macedonia; 10 National Centre of Public Health and Analyses, Directorate Mental Health and Prevention of Addictions, Acad. Ivan Evst. Geshov 15 blvd., 1431 Sofia, Bulgaria; 11 Dutch Institute for Mental Health and Addiction/Trimbos Institute, Da Costakade 45, 3521 Utrecht, VS Netherlands; 12 Present Address: INSIGHT International Institute for Mental Health and Integrated Health Systems, Cornelis Anthoniszstraat 23-1, 1071VP Amsterdam, Netherlands

**Keywords:** Community mental health services, Implementation, Collaborative Teamwork, Role perceptions

## Abstract

**Background:**

Community Mental Health Teams (CMHTs) deliver healthcare that supports the recovery of people with mental illness. The aim of this paper was to explore to what extent team members of five CMHTs newly implemented in five countries perceived that they had introduced aspects of the recovery-oriented, strength-based approach into care after a training week on recovery-oriented practice. In addition, it evaluated what the team members’ perceptions on their care roles and their level of confidence with this role were.

**Method:**

An observational intervention study using a quantitative survey that was administered among 52 health professionals (21 Nurses, 13 Psychiatrists, 9 Psychologists, 8 Social Workers) and 14 peer workers including the Recovery Self-Assessment Tool Provider Version (RSA-P), the Team Member Self-Assessment Tool (TMSA), and demographic questions was conducted. The measures were self-reported. Descriptive statistics were used to calculate the means and standard deviations for continuous variables and frequencies and percentages for categorical variables (TMSA tool and demographic data). The standard technique to calculate scale scores for each subscale of the RSA-P was used. Bivariate linear regression analyses were applied to explore the impact of predictors on the subscales of the RSA-P. Predictors with significant effects were included in multiple regression models.

**Result:**

The RSA-P showed that all teams had the perception that they provide recovery–oriented practice to a moderately high degree after a training week on recovery-oriented care (mean scores between 3.85–4.46). Health professionals with fewer years of professional experience perceived more frequently that they operated in a recovery-oriented way (*p* = 0.036, B = − 0.268). Nurses and peer workers did not feel confident or responsible to fulfil specific roles.

**Conclusion:**

The findings suggest that a one-week training session on community-based practices and collaborative teamwork may enhance recovery-oriented practice, but the role of nurses and peer workers needs further attention.

**Trial registration:**

Each trial was registered before participant enrolment in the clinicaltrials.gov database: Croatia, Zagreb (Trial Reg. No. NCT03862209); Montenegro, Kotor (Trial Reg. No. NCT03837340); Romania, Suceava (Trial Reg. No. NCT03884933); Macedonia, Skopje (Trial Reg. No. NCT03892473); Bulgaria, Sofia (Trial Reg. No. NCT03922425).

**Supplementary Information:**

The online version contains supplementary material available at 10.1186/s12888-021-03542-2.

## Background

Mental illnesses are the main cause for disability worldwide [1], and contribute substantially to loss of healthy life years [2, 3]. Moreover, people experiencing mental illnesses are more likely to be excluded from social life, are at higher risk of poverty and stigmatisation [2, 3]. They are more likely to suffer from medical comorbidity, poor physical health, and are at higher risk of premature death compared to people without mental illnesses [4]. In Europe more than one in six people have mental health problems [5], which translates to more than 21 million people currently living with depression (4.5%) and more than five million with other severe mental illnesses, such as bipolar disorder (1%) and schizophrenia (0.3%) [5].

In Europe, access to and quality of mental healthcare varies considerably across countries and systems [6]. In low- and middle-income countries between 76 and 85% of people with mental illness do not receive the treatment they need [1, 3, 7, 8]. Usual care typically involved inpatient mental healthcare and institution-based outpatient mental healthcare, without much outreach activity (i.e. home visits) or involvement of service user preferences or experiences of care. Mental health systems particularly in Eastern and Central Europe are at in the process of developing and strengthening community-based services [8, 9]. Many mental health systems have gone through a process of deinstitutionalization by replacing long-stay and institutional inpatient care with community-based alternatives [6, 10]. Shifting healthcare from the hospitals to community care aims at supporting individuals with mental illnesses in maintaining independence, promoting choices, and enhancing inclusion [10]. In Central and Eastern Europe, community-based services have often been piloted or implemented for a short-duration of time, typically in the form of a community mental health centre located in a primary care centre or a hospital [11, 12]. Sustaining the work of community-based services and ensuring that they provide a comprehensive set of services in and outside the clinic remains less common in the Central and Eastern European region.

Numerous service delivery models exist for the delivery of community-based mental health services. Often, care delivered in the community is done through a form of a community mental health team [10]. Community Mental Health Teams (CMHTs) are often multidisciplinary, including professions such as nurses, psychiatrists, psychologists, and social workers [10]. Peer workers (trained individuals with a diagnosis of severe mental illness) may be included in CMHTs as well. The combined expertise and interdisciplinary work practices of these professions enables a service user to receive more holistic care addressing their medical, mental and social needs [10]. A frequently used service delivery model and evidence-based practice for severe mental illness (SMI) is the *flexible assertive community treatment* (FACT) model [13]. The FACT model helps service user receive intensive support within their community by a multidisciplinary team, which works recovery-oriented rather than focusing on illness [14–16]. Next to a low client-staff ratio and regular team meetings, key components of the FACT model include a shared and team-based caseload, as well as home visits [13, 17]. A case manager coordinates individual caseloads, although all professionals within the team provide mental healthcare. In times of an increased need for treatment and care, the team works together to meet those needs. This care model provides an opportunity for a transition between high- and low-intensity treatment and care and a shared case-load, and thus may enhances recovery [13].

Although recovery from SMI is an individual journey, professional care and guidance can support recovery outcomes [18, 19]. A recovery-oriented approach focuses on the person, addresses stigmatisation, and facilitates social inclusion, and improves quality of life, citizenship, and participation in society [3, 20]. It is a collaborative process between mental healthcare providers and service user which facilitates shared decision making and puts the service users’ individual perspectives on recovery in the centre of the treatment [21, 22]. Recovery-oriented practice includes service user empowerment, peer support, the right to informed choice, respecting individual needs, and the right to be treated ethically [22].

Recovery usually occurs in an individual’s personal environment and includes feelings like hope, understanding of competences and skills, having an active and social life, personal autonomy, and living a meaningful life with a positive sense of self [20, 23]. Peer support in mental health is defined as support for a person with a mental health issue provided by people with personal lived experiences of a mental health issue [24]. Persons with lived experience are also increasingly embedded into service delivery teams, as a peer worker or peer specialist [25, 26]. The support of peer workers can have a positive impact on the recovery journey of people with SMI [24, 27].

Care managed by a highly qualified CMHT can support the transition from hospital to community, participation in social life and support increases in overall service user satisfaction [28–30]. This requires commitment by each team member, a shared vision, a clear distribution of roles, and a common purpose [31]. Multidisciplinary team functioning is complex and requires a deeper understanding of the different professions operating in teams, such as in CMHTs. Boundaries between the profession can act as barrier and influence communication and coordination negatively which has an impact on service user safety and care integration [32]. Previous research shows that not all healthcare providers work effectively in a multidisciplinary team [31]. Unclear role allocation, lack of clarity regarding leadership can also hinder team functioning [31].

Research has shown that comprehensive community-based mental health services can lead to an improvement in healthcare and service user outcomes, including quality of life, treatment adherence, healthcare accessibility, and social outcomes [10, 33–35]. Additionally, CMHTs can reduce the number of days spent in hospital, increase service user satisfaction, reduce suicide rates, and number of admissions to the hospital [10, 31]. Furthermore, most people with SMI favour recovery-oriented healthcare services provided in their community in order to participate in social life and sustain employment [23, 28, 29].

These insights have led to the design and implementation of CMHTs for providing inclusive mental healthcare based on the recovery-oriented approach and social support in five sites in five Central and Eastern European countries within the *Large-scale implementation of community-based mental health care for people with severe and enduring mental ill health in Europe* project (RECOVER-E) [36]. The ultimate aim of the RECOVER-E project is to implement and evaluate multidisciplinary CMHTs delivering care in a recovery-oriented way to people with SMI and compare it to the treatment as usual on three levels: 1) service user outcome, 2) performance of team members; and 3) socioeconomic evaluation [36].

The present study aim is two-fold: to explore the extent to which CMHT members perceive they deliver care in a recovery-oriented, strength-based approach, and to evaluate team members’ perceptions on their care roles in the CMHT and their level of confidence with this role.

## Methods and design

### Study Design

The research presented in this manuscript is part of the RECOVER-E project, a European research project with five randomized trials on the implementation of community-based mental healthcare for people with SMI [36]. In each of the five sites, the study was designed as a clinical and health-economic evaluation on the basis of a hybrid effectiveness-implementation trial, which assesses both implementation outcomes and service user health outcomes. Each of the five hybrid trials is conducted as pragmatic randomised trial in two parallel groups with measurements among service users and healthcare providers (members of the CMHTs within each site).

The presented study focuses on the fidelity of the CMHT (whether the model of community mental healthcare had been adopted as planned) and the experiences of the involved health workers. It reports the findings from a paper-based survey among all team members of the CMHTs in each project site at local start of the study, after having completed a one-week training session on the concept of CMHT and the principles of recovery-oriented care. The local research teams were responsible for distributing the survey to the CMHT members after the training.

### Study setting

Community mental health teams were established and implemented in Sofia, Bulgaria; Zagreb, Croatia; Skopje, North Macedonia; Kotor, Montenegro; Siret-Suceava, Romania (Supplementary Material). The full rationale and selection criteria for these sites is described in a prior publication [36].

### Participants

This study included all members of the CMHTs in the five sites. Each team consists of at least one nurse, psychiatrist, psychologist, social worker, and a peer worker (person with lived experience of a SMI), all 18 years of age or older who also are members of the CMHT. Peer workers are existing service user with a diagnosis of a SMI at each of the mental health services in the 5 sites. All teams were newly established for the RECOVER-E project, but some team members had collaborated in teams in previous projects. All health professionals and peer workers were required to provide consent to participate in the study. Informed consent was obtained by each health professional and each peer worker verbally.

### Sampling and recruitment

#### Sampling and recruitment were organised by the local research team in each project site individually

##### Zagreb, Croatia

During a daily meeting at the department of psychiatry at the University Hospital Centre Zagreb (ZUHC) the RECOVER-E project and its goals were presented. Healthcare professionals who were interested in this topic could approach the local principal investigator and decide if they want to participate. All healthcare professionals who accepted to become a part of the CMHT participated in this study, and were employees of the ZUHC. Peer workers were recruited from patients that were treated at the ZUHC due to SMI and were recovered during psychosocial treatment afterwards.

##### Kotor, Montenegro

In Montenegro CMHT members were employed by the Special Psychiatric Hospital in Kotor. They have been selected by the director of the clinic based on their commitment to work, their previous work results and on their own motivation and interest to work in this kind of programme and their awareness of the need to provide more than just hospital-based services for service users. The directors explained the project and the nature of the study and asked if they want to participate on a voluntary basis. In early October 2018 three peer workers were recruited from a group of locally treated clients at the hospital and the Mental Health Centre in Kotor. Inclusion criteria were a presence of severe mental health disorder, the willingness to participate in working activities of mental health team, and the capacity to offer peer support based on the opinion of the treating psychiatrist. All healthcare proffesionals and peer workers who accepted to become a part of the CMHT participated in this study.

##### Siret, Romania

In Romania CMHT members were selected out of the people working at the hospital in Siret by the local principle investigator. All team members were willing to spend some of their free time on pioneering a new service in mental health. Moreover, they were the most active during the on-site training provided by the project coordination. The peer workers were selected together by the social worker, psychiatrist and psychologist based on their professional experience. All healthcare professionals and the peer workers who accepted to become a part of the CMHT participated in this study.

##### Skopje, North Macedonia

All CMHT members were employed at the University Clinic of Psychiatry Skopje and were recruited by the directors of the clinic. They selected the employees based on their willingness and motivation to try something new. The directors explained the project and the nature of the study and asked if they want to participate. The two peer workers, who were recruited, were treated at the clinic due to SMI previously and were recovered during psychosocial treatment. They are not employed at the clinic but were also selected by the directors of the clinic. Everybody took part in the first training that was organized in Skopje. All healthcare professionals and the peer workers who accepted to become a part of the CMHT also decided to participate in this study. Six members went to the second training in the Netherlands and updated the others.

##### Sofia, Bulgaria

Healthcare providers were recruited by the director of the MHC Shipkovenski. They decided on a voluntary basis if they want to participate in this project. All CMHT members were employed at the MHC “Prof. N. Shipkovenski” a hospital for the district Sofia. The psychiatrists who work at the MHC Shipkovenski and those who collaborate with the centre suggested service user who were interested to fill in the role as peer workers. The director of the MHC Shipkovenski selected ten potential participants which participated in the training in July 2019. Three of them were invited and agreed to participate in the project. All healthcare professionals and the peer workers who accepted to become a part of the CMHT also decided to participate in this study.

### Training on recovery-oriented practices

After members of the CMHTs were appointed by each mental health service in the five sites, CMHT members participated in a two-week training programme, with one week of the training carried out in their home country and one week as an intensive site visit and training week in the Netherlands, hosted by GGZ Noord-Holland-Noord. Data was collected after the first training week. In this training, healthcare professionals and peer workers had the chance to improve their understanding of community based mental health approaches, with the aim of being able to implement a cohesive team in their city or district. The training programme for the CMHT were developed by a multidisciplinary Expert Panel (including a peer worker) and reviewed closely by the implementation site coordinators for local relevance and adaptation. The training covered key components of community mental health care, working with a shared caseload, and home treatments. It focused on building the hands-on skills and competencies necessary for delivering high quality community care, as well as working with peer specialists and families. A substantial component of the training (and subsequent mentoring and hands-on coaching) focused on the most difficult change process in building a sustainable CMHT, which is changing the mindset from viewing treatment for people with SMI as custodial (protection from communities/societies, care in institutions and hospitals) and shift to perceiving services as an aid to a meaningful life in the community [37].

### Data collection

Data was collected using a paper-based survey administered among CMHT members at the local start of the study during a staff meeting in each site by the local research team after the first week of training in their home country (Table 1). The questionnaire items had been translated prior to the start of the study at each site into the local language by members of the local research team who were fluent in English and in the local language and back translated. It took the team members approximately 15 to 20 min to complete the questionnaire. An informed consent was signed by all selected staff members prior to the start of the study.

**Table 1:** Period of data collection and dates of the two training weeks per project site

	Zagreb, Croatia	Kotor, Montenegro	Siret, Romania	Skopje, North Macedonia	Sofia, Bulgaria
Period of Recruitment	May 2018 – September 2018	1. – 15. October 2018	January 2018 and March 2018	20. – 31. May 2019	April 2019 – June 2019
Training Week 1 (on-site-training)	24. – 28. September 2018	4. – 8. November 2018	28. January – 02. February 2019	24. – 28. June 2019	15. – 19. July 2019
Data collection	February 2019	February 2019	April 2019	June 2019	November 2019
Training Week 2 (Netherlands)	6. – 10. May 2019	6. – 10. May 2019	6. – 10. May 2019	02. – 06. December 2019	02. – 06. December 2019

### Measurements

#### Sociodemographic data questionnaire

The sociodemographic part of the questionnaire included questions such as year of birth, sex, profession, years of professional experience, and place of work.

#### Recovery Self-Assessment (RSA-Provider Version)

The Recovery Self-Assessment (RSA) is a 32-item measure developed to assess to what degree a program implements recovery-oriented practice [38]. It is a self-reflective tool that is designed to identify efforts made by healthcare agencies to provided recovery-oriented care. Research has shown that the RSA has moderate to strong internal reliability (Cronbach’s α 0.63–0.90) [38–41]. Response options include a 5-point Likert scale from 1 (strongly disagree) to 5 (strongly agree) and two additional answering options (Don’t know and Not Applicable) [42]. There are four different versions of the RSA: Person in recovery, Family member/advocate, provider, and CEOs/Directors. In this study the provider version (RSA-P) was used. The measure covers the following five subscales: Life Goals, Involvement, Diversity of Treatment Options, Choice, and Individually Tailored Services. The Life Goal domain refers to the extent to which staff helps with the development and achievement of life goals based on the preferences of the patient. Involvement indicates to what degree healthcare provider and clients perceive that clients are involved in their healthcare and in decision-making. Diversity of Treatment Option Subscale refers to what extend the healthcare organisation provides different treatment options and supports clients to get involved in non-mental health activities. The Choice domain indicates to what degree healthcare provider and clients feel that choices are available to clients and if the choice is respected. The subscale Individually Tailored Services refers to the perception that healthcare services are tailored to individuals’ personal needs, culture and affectations [41]. Higher scores indicate a greater degree of implementation of recovery-oriented practices [38].

#### Team Member Self-Assessment

The Team member Self-Assessment Tool (TMSA) developed by the Advancing Integrated Mental Health Solutions (AIMS) Centre of the University of Washington, Psychiatry & Behavioural Sciences Division of Population Health and is a part of the Team building and workflow guide [43]. The tool (worksheet) consists of 26-items that allow each member of a care team to think about what collaborative care roles he/she currently practices. The worksheet includes five different care roles: Identify and Engage Patients, Track Treatment Outcome, Initiate, and Provide Treatment, Proactively Adjust Treatment if Patients are not responding, and other tasks Important for our Program. Response options for the first question “*Is this your role*” include yes or no. Answering option for the second question “*Your level of comfort with this task*” include high or medium/low.

### Data analysis

All questionnaires were included in the analysis. Prior to analysis, all variables were checked for data entry errors and missing values. The two response options (‘Don’t Know’ and ‘Not Applicable’) were set as user missing value when conducting the first analysis since this is a common method for categorial variables with response options like ‘Not Applicable’ or ‘Don’t Know’. Then the standard technique to calculate scale scores for each subscale of the RSA-P was used [44]. This meant that, for each subscale all associate items were summarized and aggregate measures were constructed for further analysis for the whole sample as well as per project site. This method only allows for a few missing values. Thus, items with a high number (*n* > 6) of ‘Not Applicable’ responses were reviewed and discussed with the local research teams of each site to understand in what way this response option was interpreted by the CMHT members who completed the questionnaire. Most of these items turned out to be relating to services/treatment options which were not offered by the hospitals, the mental health institutes or not within the scope of the RECOVER-E project. Thus, the answering option ‘Not Applicable’ was combined with ‘Strongly Disagree’ in the second and final analysis, as both indicated that those services were not provided. Bivariate linear regression analyses were applied to explore the impact of predictors on the five aggregate measures for the whole sample. Predictors with significant effects were included in multiple regression models, albeit these were considered as highly tentative given the small sample size. The internal consistency of the RSA-P and its five subscales were evaluated using Cronbach’s alpha coefficient.

Descriptive statistics were used to calculate the means and standard deviations for continuous variables and frequencies and percentages for categorical variables for the TMSA tool and demographic data. For interpretation of the findings the cut-off points for the TMSA tool for each profession was 50% indicated that half of the given profession felt like a listed role is their responsibility or that half of them felt confident to fulfil these roles. Two participants indicated ‘other’ as profession and were excluded from the TMSA analysis. Data was analysed with the Statistical Package for Social Science SPSS version 25 [45]. Statistical significance was defined as *p* < 0.05.

### Ethical considerations

Prior to the start of the study in August 2018, ethical approval was obtained from the Medical Ethics Committee of the Heidelberg School of Medicine (S-496/2018), which led the RECOVER-E project work package for research evaluation and is responsible for the overall coordination of the project’s research activities. In addition, each study at each implementation site received ethical approval from the local institutional review board prior to the start of the study. Before consent was given by the participants, a member of the local research team at each project site explained the aims and objectives of the study.

The study was performed in accordance with the ethical standards as laid down in the 1964 Declaration of Helsinki and its later amendments.

#### Siret, Romania; Sofia, Bulgaria; Zagreb, Croatia

The local research team provided verbal information about the project to participating health professionals and peer workers. Verbal consent was obtained from each health professional and peer worker prior to the start of the study.

#### Skopje, North Macedonia

Eligible health professionals and peers were informed verbally by the local research team. Health professionals and peers who chose to participate in the CMTH received an informed consent letter and gave their verbal consent to participate, prior to the start of the study.

#### Kotor, Montenegro

Informed consent of all eligible health professionals and peer workers who decided to take part in this study was obtained in form of a signed informed consent form. The purpose and the aim of the study was explained to them prior to the study verbally.

## Results

### Description of the sample

All team members from each site filled in the questionnaire (*n* = 66, 100%). Table 2 presents the descriptive characteristics of the sample categorised by countries. The largest community mental health team was the Croatian team with *n* = 21 (*n* = 21, 31.8% of the total sample), the smallest team was the Romanian team with *n* = 6 (*n =* 6, 9.1% of the sample) individuals. Of the total study population 65.2% (*n =* 43, 62.2%) were female. The mean age was 40.02 (SD = 10.96) years. A third of the participating professionals were nurses (*n =* 21, 31.8%). A vast majority (*n* = 42, 63.6%) of the healthcare professionals had more than 5 years of experience.

**Table 2:** Description of the study population (*n* = 52 healthcare professionals and *n* = 14 peer workers) per project site

Project site	Zagreb, Croatia ***n =*** 21	Kotor, Montenegro ***n =*** 15	Siret, Romania ***n =*** 6	Skopje, North Macedonia ***n =*** 10	Sofia, Bulgaria ***n =*** 14	Total ***N =*** 66
**Sex**						
Female	15 (71.4%)	9 (60.0%)	4 (66.7%)	5 (50.0%)	10 (71.4%)	43 (65.2%)
Male	6 (28.6%)	6 (40.0%)	2 (33.3%)	5 (50.0%)	4 (28,6%)	23 (34.8%)
**Age**						
Mean (SD)	41.3 (12.56)	38.47 (9.71)	43.67 (6.98)	39.10 (11.22)	38.93 (11.83)	40.02 (10.96)
**Profession**						
Nurse	9 (42.9%)	5 (33.3%)	2 (33.3%)	2 (20.0%)	3 (21.4%)	21 (31.8%)
Psychiatrist	4 (19.0%)	4 (26.7%)	1 (16.7%)	2 (20.0%)	2 (14.3%)	13 (19.7%)
Psychologist	1 (4.8%)	2 (13.3%)	1 (16.7%)	2 (20.0%)	3 (21.4%)	9 (13.6%)
Social worker	3 (14.3%)	1 (6.7%)	1 (16.7%)	1 (10.0%)	2 (14.3%)	8 (12.1%)
Peer worker	4 (19.0%)	3 (20.0%)	1 (16.7%)	2 (20.0%)	4 (28.6%)	14 (21.2%)
Other	0	0	0	1 (10.0%)	0	1 (1.5%)
**Professional Experience**						
Less than one year	0	2 (13.3%)	1 (16.7%)	1 (10.0%)	4 (28.6%)	8 (12.1%)
Between one and two years	1 (4.8%)	0	0	1 (10.0%)	3 (21.4%)	5 (7.6%)
Between two and three years	3 (14.3%)	0	0	0	0	3 (4.5%)
Between three and four years	2 (9.5%)	0	1 (16.7%)	1 (10.0%)	0	4 (6.1%)
Between four and five years	1 (4.8%)	0	0	3 (30.0%)	0	4 (6.1%)
More than five years	14 (66.7%)	13 (86.7%)	4 (66.7%)	4 (40.0%)	7 (50.0%)	42 (63.6%)

*Team size varies due different local human resources

### Recovery Self-Assessment Provider Version (RSA-P)

#### Overall Recovery Self-Assessment Scale

The members of the CMHT in Romania and Bulgaria reported the highest mean scores of the RSA-P subscales, with an overall scale 4.46 (SD 0.327) and 4.46 (SD 0.211), respectively. The team members of the Croatian team reported an overall mean of 4.16 (SD 0.404). The lowest means were indicated by the Macedonian team 4.05 (SD 0.694) and the Montenegrin team 3.58 (SD 0.275). This pattern was similar for the different subscales (Table 3).

**Table 3:** Recovery Self-Assessment Scale (Provider Version) per project site

Project sites					
RSA-P subscale, mean score (standard deviation)	Zagreb, Croatia (***n =*** 21)	Kotor, Montenegro (***n =*** 15)	Siret, Romania (***n =*** 9)	Skopje, Macedonia (***n =*** 10)	Sofia, Bulgaria (***n =*** 14)
**Life Goals**	4.1 (0.46)	3.7 (0.33)	4.6 (0.37)	4.0 (0.68)	4.4 (0.28)
**Involvement**	3.8 (0.57)	2.7 (0.65)	4.6 (0.45)	4.0 (0.88)	4.5 (0.31)
**Diversity of treatment option**	4.2 (0.54)	3.1 (0.23)	4.6 (0.36)	4.1 (0.75)	4.7 (0.19)
**Choice**	4.4 (0.43)	4.5 (0.32)	4.5 (0.35)	4.0 (0.72)	4.8 (0.15)
**Individually tailored service**	4.0 (0.46)	3.4 (0.47)	4.7 (0.37)	4.1 (0.91)	4.6 (0.24)
**Total RSA-P**	4.2 (0.40)	3.6 (0.28)	4.5 (0.33)	4.1 (0.69)	4.5 (0.21)

* Response options include a 5-point Likert scale from 1 (strongly disagree) to 5 (strongly agree) and two additional answering options (Don’t know and Not Applicable, Higher scores indicate a greater degree of implementation of recovery-oriented practices

#### Associations between individual characteristics and Recovery Self-Assessment

Profession (*p* = 0.029, beta coefficient = 0.271) and professional experience (*p* = 0.008, beta coefficient = − 0.328) were associated with the total RSA-P score. However, only professional experience (*p* = 0.036, beta coefficient = − 0.268) remained significant in the multiple regression analysis with both predictors. The effect of professional experience on the total RSA-P implied that healthcare providers with fewer years of professional experience (less than 5 years) were more likely to perceive that their healthcare practice operated recovery-oriented compared to team members with more years of professional experience.

##### RSA-P-Subscales

The amount of professional experience had an impact on the degree to what healthcare provider perceived choices are available to clients and if these choices are respected (*p* = 0.027, beta coefficient = − 0.286). More specifically, professionals with fewer years of professional experience (less than 5 years) were more likely to perceive that individual choices are available to clients and that these choices are respected than with team members with more years of work experience. Additionally, work experience (*p* = 0.003, beta coefficient = − 0.357) and profession (*p* = 0.012, beta coefficient = 0.311) were associated with the extent to which staff helps with the development and achievement of life goals based on the preferences of the patients. In the multiple regression analysis with both predictors, only professional experience remained significant (*p* = 0.023, beta coefficient = − 0.286,). Thus, CMHT members with more years of professional experience (more than 5 years) perceived that their role was less supportive regarding helping clients with achieving and developing their individual life goals. Healthcare provider with less work experience (less than 5 years) had a higher tendency to perceive that clients are involved in their healthcare and in decision-making compared to their those with more years of experience (*p* = 0.016, beta coefficient = − 0.311). Professionals with more years of work experience (more than 5 years) felt less like their healthcare organisation provides different treatment options or supports clients to get involved in non-mental health activities (*p* = 0.017, beta coefficient = − 0.302). The expected effect of professional experience on the subscale Individually Tailored Services was not significant although close (*p* = 0.051, beta coefficient = − 0.251).

#### Team Member Self-Assessment

The findings of the TMSA are reported by professions. More than 50% indicate that most members of a profession saw themselves in a certain role and/or felt highly confident in fulfilling these roles. The questionnaire was completed after the healthcare professionals received the first week of training and started to work as CMHTs.

##### Nurses

A large majority of the nurses (81.0%) saw themselves in the role of identifying and engaging patients. Nevertheless, only a small number (33.3%) stated that they feel highly confident to fulfil the different responsibilities within this role on the CMHT. A third stated that diagnosing behavioural health disorders is their responsibility (33.3%). Over 50% also saw themselves as having a role in tracking treatment outcomes, although only a few felt confident to fulfil this role. A vast majority of nurses saw their role in different responsibilities within this role as well, such as by conducting behavioural health assessments, developing, and updating behavioural health treatment plans, educating service user about symptoms and treatment option, brief counselling, activity scheduling, behavioural activation, facilitating referral to specialty care or social services, and creating and supporting relapse prevention plan. However, only a few felt confident to fulfil these responsibilities. A large majority of nurses also stated that proactively adjust treatment if patients are not responding is their role, however less than a third of them felt confident to fulfil these roles. Although other tasks such as administrative support for program (scheduling, resources) is a role more than 50% of the nurses also saw themselves in, only a few felt confident to fulfil these roles (Tables 4, 5 and 6).

**Table 4:** Team Member Self-Assessment tool: Identify and Engage Patients and Track Treatment Outcome

In total ***n*** = 65 professionals		Nurse ***n*** = 21	Psychiatrist ***n*** = 13	Psychologist ***n*** = 9	Social worker ***n*** = 8	Peer worker ***n*** = 14
**Identify and Engage Patients**						
Identify People who may need help	Is this my role^a^	17 (81.0%)	13 (100.0%)	8 (88.9%)	8 (100.0%)	3 (21.4%)
	Highly confident^b^	7 (33.3%)	13 (100.0%)	8 (88.9%)	6 (75.0%)	2 (14.2%)
Screen for behavioural health problems using valid measures	Is this my role^a^	13 (61.9%)	12 (92.3%)	8 (88.9%)	5 (62.5%)	1 (7.1%)
	Highly confident^b^	5 (23.8%)	11 (84.6%)	6 (66.7%)	2 (25.0%)	1 (7.1%)
Diagnose behavioural health disorders	Is this my role^a^	7 (33.3%)	13 (100.0%)	7 (77.7%)	5 (62.5%)	1 (7.1%)
	Highly confident^b^	5 (23.8%)	12 (92.3%)	6 (66.7%)	1 (12.5%)	0 (0.0%)
Engage patients in collaborative care program and introduce care team	Is this my role^a^	17 (81.0%)	13 (100.0%)	7 (77.7%)	8 (100.0%)	11 (78.6%)
	Highly confident^b^	10 (47.6%)	10 (76.9%)	4 (44.4%)	4 (50.0%)	4 (28.6%)
**Track Treatment Outcome**						
Track treatment engagement & adherence using registry	Is this my role^a^	15 (71.4%)	12 (92.3%)	5 (55.6%)	5 (62.5%)	8 (57.1%)
	Highly confident^b^	3 (14.3%)	9 (69.2%)	1 (11.1%)	2 (25.0%)	1 (7.1%)
Reach out to patients who are non-adherent or disengaged	Is this my role^a^	17 (81.0%)	12 (92.3%)	8 (88.9%)	6 (75.0%)	9 (64.3%)
	Highly confident^b^	6 (28.6%)	8 (61.5%)	3 (33.3%)	2 (25.0%)	1 (7.7%)
Track patients’ symptoms with measurement tool	Is this my role^a^	12 (57.1%)	12 (92.3%)	8 (88.9%)	5 (62.5%)	4 (28.6%)
	Highly confident^b^	5 (28.8%)	9 (69.2%)	5 (55.5%)	3 (37.5%)	0 (0.0%)
Track medication side effects and concerns	Is this my role^a^	13 (61.9%)	13 (100.0%)	4 (44.4%)	3 (37.5%)	7 (50.0%)
	Highly confident^b^	9 (42.9%)	11 (84.6%)	1 (11.1%)	1 (12.5%)	1 (7.1%)
Track outcome of referrals and other treatments	Is this my role^a^	15 (71.4%)	13 (100.0%)	7 (77.7%)	8 (100.0%)	8 (57.1%)
	Highly confident^b^	8 (38.1%)	11 (84.6%)	4 (44.4%)	2 (25.0%)	2 (14.3%)

Absolute Numbers; ^a^ Answering categories were: yes; no; ^b^ Answering categories were: high; Med/low confident; ‘other *n* = 1’ was excluded from this analysis

**Table 5:** Team Member Self-Assessment tool: Initiate and provide treatment

In total ***n =*** 65 professionals		Nurse ***n =*** 21	Psychiatrist ***n =*** 13	Psychologist ***n =*** 9	Social worker ***n =*** 8	Peer worker ***n =*** 14
**Initiate and provide treatment**						
Perform behavioural health assessment	Is this my role^a^	12 (57.1%)	13 (100.0%)	8 (88.9%)	6 (75.0%)	8 (57.1%)
	Highly confident^b^	5 (28.8%)	11 (84.6%)	5 (55.6%)	3 (37.5%)	2 (14.3%)
Develop and update behavioural health treatment plan	Is this my role^a^	12 (57.1%)	13 (100.0%)	8 (88.9%)	5 (62.5%)	10 (71.4%)
	Highly confident^b^	3 (14.3%)	11 (84.6%)	3 (33.3%)	2 (25.0%)	2 (14.3%)
Service user education about symptoms and treatment option	Is this my role^a^	14 (66.7%)	13 (100.0%)	8 (88.9%)	4 (50.0%)	9 (64.3%)
	Highly confident^b^	7 (33.3%)	13 (100.0%)	6 (66.7%)	4 (50.0%)	8 (57.1%)
Prescribe psychotropic medications	Is this my role^a^	4 (19.0%)	13 (100.0%)	1 (11.1%)	1 (12.5%)	1 (7.1%)
	Highly confident^b^	1 (4.8%)	13 (100.0%)	1 (11.1%)	0 (0.0%)	0 (0.0%)
Service user education about medications and side effects	Is this my role^a^	6 (28.6%)	13 (100.0%)	2 (22.2%)	1 (12.5%)	4 (28.6%)
	Highly confident^b^	4 (19.0%)	13 (100.0%)	2 (22.2%)	0 (0.0%)	1 (7.1%)
Brief counselling, activity scheduling, behavioural activation	Is this my role^a^	13 (61.9%)	13 (100.0%)	9 (100.0%)	7 (87.5%)	8 (57.1%)
	Highly confident^b^	7 (33.3%)	11 (84.6%)	6 (66.7%)	5 (62.5%)	2 (14.3%)
Evidence-based psychotherapy	Is this my role^a^	6 (28.6%)	11 (84.6%)	8 (88.9%)	3 (37.5%)	1 (7.1%)
	Highly confident^b^	4 (21.1%)	8 (61.5%)	6 (66.7%)	1 (12.5%)	0 (0.0%)
Identify and treat coexisting medical conditions	Is this my role^a^	7 (33.3%)	13 (100.0%)	3 (33.3%)	1 (12.5%)	0 (0.0%)
	Highly confident^b^	4 (21.1%)	11 (84.6%)	3 (3339%)	0 (0.0%)	0 (0.0%)
Facilitate referral to specialty care or social services	Is this my role^a^	13 (61.9%)	13 (100.0%)	4 (44.4%)	8 (100.0%)	2 (14.3%)
	Highly confident^b^	5 (28.8%)	11 (84.6%)	2 (22.2%)	7 (87.5%)	1 (7.1%)
Create and support relapse prevention plan	Is this my role^a^	17 (81.0%)	13 (100.0%)	9 (100.0%)	8 (100.0%)	5 (35.7%)
	Highly confident^b^	8 (38.1%)	13 (100.0%)	6 (66.7%)	5 (62.5%)	1 (7.1%)

Absolute Numbers; ^a^ Answering categories were: yes; no; ^b^ Answering categories were: high; Med/low confident; ‘other *n* = 1’ was excluded from this analysis

**Table 6:** Team Member Self-Assessment tool: Proactively adjust treatment if patients are not responding and other tasks important for our project

In total ***n =*** 65 professionals		Nurse ***n =*** 21	Psychiatrist ***n =*** 13	Psychologist ***n =*** 9	Social worker ***n =*** 8	Peer worker ***n =*** 14
**Proactively adjust treatment if patients are not responding**						
Assess need for changes in treatment	Is this my role^a^	15 (71.4%)	13 (100.0%)	7 (77.8%)	7 (87.5%)	5 (35.7%)
	Highly confident^b^	4 (21.1%)	12 (92.3%)	4 (44.4%)	5 (62.5%)	0 (0.0%)
Facilitate changes in treatment/ treatment plan	Is this my role^a^	15 (71.4%)	13 (100.0%)	9 (100.0%)	7 (87.5%)	5 (35.7%)
	Highly confident^b^	5 (28.8%)	10 (76.9%)	4 (44.4%)	4 (50.0%)	0 (0.0%)
Provide caseload-focused psychiatric consultation	Is this my role^a^	13 (61.9%)	13 (100.0%)	4 (44.4%)	5 (62.5%)	6 (42.9%)
	Highly confident^b^	6 (28.6%)	11 (84.6%)	0 (0.0%)	2 (5.0%)	2 (14.3%)
Provide in-person psychiatric assessment when needed	Is this my role^a^	12 (57.1%)	13 (100.0%)	4 (44.4%)	1 (12.5%)	1 (7.1%)
	Highly confident^b^	5 (23.8%)	13 (100.0%)	0 (0.0%)	1 (12.5%)	1 (7.1%)
**Other tasks important for this program**						
Coordinate communication among team members/ providers	Is this my role^a^	11 (52.4%)	11 (84.6%)	8 (88.9%)	5 (62.5%)	7 (50.0%)
	Highly confident^b^	4 (21.1%)	9 (69.2%)	5 (55.6%)	3 (37.5%)	3 (21.4%)
Administrative support for program (e.g., scheduling, resources)	Is this my role^a^	15 (71.4%)	11 (84.6%)	6 (66.7%)	7 (87.5%)	7 (50.0%)
	Highly confident^b^	4 (21.1%)	9 (69.2%)	3 (33.3%)	4 (50.0%)	2 (14.3%)
Clinical supervision for Program	Is this my role^a^	10 (47.6%)	12 (92.3%)	5 (55.6%)	4 (50.0%)	7 (50.0%)
	Highly confident^b^	5 (23.8%)	12 (92.3%)	3 (33.3%)	1 (12.5%)	3 (21.4%)

Absolute Numbers; ^a^ Answering categories were: yes; no; ^b^ Answering categories were: high; Med/low confident; ‘other *n* = 1’ was excluded from this analysis

##### Psychiatrists

Most psychiatrists saw themselves in all listed roles, and also felt confident to fulfil these roles. They particularly identified with the role of tracking treatment outcomes and initiating and providing treatment (Tables 4, 5 and 6).

##### Psychologists

A vast majority of psychologists identified with all roles listed and felt highly confident to fulfil these roles. Although they felt like they were responsible for some tasks such as prescribing psychotropic medications, service user education about medications and side effects, identifying and treating coexisting medical conditions, and facilitating referral to specialty care or social services of within the role of tracking treatment outcomes, they saw themselves more in the roles of identifying and engaging patients and initiating and providing treatment (Tables 4, 5 and 6).

##### Social workers

A majority of social workers saw themselves in all roles listed, but did not feel confident to fulfil these roles. All social workers stated that they were responsible for identifying people who may need help, engaging patients and introducing the care team to the patient. Interestingly, all social workers saw themselves in the role of tracking outcome of referrals and other treatments, but only two felt confident to fulfil this role (25.0%). Initiate and provide treatment is a role mainly psychiatrists identified with; however, all social workers stated that they saw themselves in facilitate referral to specialty care or social services and create and support relapse prevention plans, and indicated feeling highly confident to fulfil these tasks (Tables 4, 5 and 6).

##### Peer workers

Almost all peer workers saw themselves in the role of engaging service users in the care program and introduce care team to the service user (78.6%), only around a third felt confident to fulfil this role (28.6%). Interestingly, more than half of peer workers saw themselves in the role of tracking treatment outcomes, except for tracking patients’ symptoms with measurement tools, although they did not feel confident to fulfil this role. They also stated to be responsible for some tasks within the role of initiating and providing treatment like performing behavioural health assessment, develop and update behavioural health treatment plan, service user education about symptoms and treatment option, and brief counselling, activity scheduling, behavioural activation. However, they only felt confident to fulfil the task of educating patients about symptoms and treatment options. Less than 50% felt responsible for the role of proactively adjusting treatment if patients are not responding. Half of the peer workers identified with the role of fulfilling other tasks important for the program, nevertheless only a few felt confident enough to fulfil this role (Tables 4, 5 and 6).

## Discussion

The aim of this study was to explore the extent to which CMHT members perceived that they deliver care in a recovery-oriented, strength-based approach after they received the first week of a two-weeks of training session covering the concept of recovery-oriented practice and started to work as CMHT. We found that all CMHT members in the five sites perceived that they incorporated recovery principles (such as involving service user in the management of their own care, giving them a voice and a choice as well as tailoring the service they provide to the need of the individuals) into their practice to a moderately high degree based on the RSA-P. Specific aspects such as connecting service user with self-help, peer support or advocacy groups and programs, and embedding service user on advisory boards and management meetings showed room for enhanced implementation. Healthcare professionals with less professional experience had the tendency to perceive that their practice implemented recovery-oriented care principles compared to team members with a higher degree of professional experience.

The importance of shifting towards placing the service user at the centre of care and embedding recovery principles into clinical practice has been recognised in international documents such as the WHO Mental Health Action Plan [46]. Globally, mental professional organizations and mental health systems have introduced curricula or training on recovery-oriented care, such as the Substance Abuse and Mental Health Services Administration (SAMHSA) recovery-oriented curricula introduced in 2009 [47]. CMHT members perceived that they already embedded recovery-oriented care principles into their clinical practice; however, CMHT members in this study are a group of highly motivated health care professionals within the mental health workforce in each of the five sites, who are committed to furthering their care delivery strategies and improving client outcomes. They are therefore not represented of the typical mental health workforce, and it is possible that the motivation to provide recovery-oriented care within the teams is higher compared to healthcare providers who have not been selected or are not interested in community care. It is possible that younger generations of health professionals are more motivated to adopt new methods in care delivery and client communication. These findings are consistent with those by Malone et al. [10]. They conducted a systematic review on the effects of CMHT management compared to non-research CMHT management. All healthcare professional working as CMHT included in their review have been selected or linked to the research programme. Hence, it is not clear if the results can be generalised from selected or highly committed professionals to non-selected professionals. The same applies to the findings of this study. The members of each CMHT of this study attended the first week of two-weeks training on the concept of recovery provided by the coordinating project team. This training might have a positive impact on the perceptions of the healthcare provider regarding the degree of recovery-oriented care within their organisation. Prior research shows that new knowledge and skills can enhance motivation and enthusiasm among health care professionals. One prior study found that staff which had been trained in recovery-oriented interventions had higher perceptions of recovery-oriented care compared to nontrained staff [22]. On its own, training cannot sustain motivation and shifts in practices, and other implementation strategies such as mentoring, supervision and feedback [22, 48], and booster training [49] focused on knowledge acquisition and skill-building are important for sustaining practice changes in community mental health care [50].

Related to our second aim to explore how CMHT members perceived their new roles within a CMHT format and as a multidisciplinary team, we found that nurses saw themselves mainly in the role of identifying and engaging patients, tracking treatment outcomes, proactively adjusting treatment if patients are not responding and other tasks important for this project. Nevertheless, compared to the psychiatrists and psychologists, most of the nurses did not feel confident to fulfil specific roles. Psychiatrists, psychologists, and social workers saw themselves in almost all roles listed. In comparison with the psychologists and social workers, psychiatrists felt highly confident to fulfil each role. Peer workers mainly saw themselves in the role of engaging patients and fulfil other tasks important to the project. Nevertheless, they did not feel confident to fulfil specific roles. Interestingly, most peer workers saw themselves in roles included within the domain of track treatment outcomes and initiate and provide treatment. CMHT members of this study, especially nurses and peer workers, are not always aware of their role or responsibilities or do not feel confident to fulfil specific roles. It makes sense that for example nurses do not feel confident doing some of the tasks that a psychiatrist usually does, as it is not necessarily their role in the teams, it may be something that they have not been trained for and/or it is something that they are legally not allowed to. Moreover, it may be due to the specific training/education these professionals acquire through their university career as well as longstanding traditions. In Croatia for example university level education of nurses is very new [51]. Thus, nurses may have not yet acquired their professional identity. This might have an impact on how nurses identified with certain roles and responsibilities. Additionally, peer workers, in general, do not have any specific peer support education to define their professional identity. This findings are consistent with those by Carpenter et al. [52]; in their study they investigated the impact of working in multidisciplinary CMHTs in North England on social workers and health professionals. They particularly examined the relationship between team identification, team functioning, psychological well-being and job satisfaction [52]. Although they found out that there is a moderate to high level of role clarity within the members of CMHTs, various role conflicts were detected [52]. Conflicts included disagreements between professions or discipline, workload, misunderstandings of roles and responsibilities or an increase in paperwork [52]. Carpenter et al. [52] concluded that role clarity promoted job satisfaction and decreases work related stress. In our study role clarity seems to be problematic, thus conflicts and disagreements may be pre-programmed. In addition, psychiatrists, psychologist, and social workers saw themselves in almost all listed roles, regardless of being confident to fulfil those roles, an unclear division of roles impeded effective team collaboration and cooperation. The existing stigmatisation in the society and among healthcare professionals may had an impact on the role of peer workers. The fact that peer workers are more and more involved in providing mental health care requires a huge shift in the way mental health professionals think. Additionally, the level of self-stigmatisation among peers may be very high and influencing these results of this study as well [53, 54].

Another source of role conflict detected by Carpenter et al. [52] was inadequate allocation of resources for people with SMI e.g. access to appropriate service based on individual needs. These findings agree with those by Singh [31], in his study he reported that the rapid reduction of beds for people with SMI had an negative impact on the care provided by CMHTs. Although CMHTs may operate effectively, a majority of patients with SMI need to be admitted to acute hospital care to receive the care they need [31]. Thus, access to an adequate number of acute beds and hospital care is necessary not only for patients with SMI but also for CMHTs to provide effective community-based mental health care and to avoid conflicts.

### Implications for practice and policy

- It takes time to adjust to new roles and responsibilities when working in a new service delivery format, such as a community mental health team. Role clarification and implementation strategies that span beyond training alone are needed to enhance teamwork and role confidence.
- Moving towards embedding more recovery-oriented principles in clinical practice takes time and practice. Training can help to raise enthusiasm for this process of change, however more practice and time is needed
- It is meaningful to capture changes in perceptions and experiences in role changes in care teams over time, and tailor implementation strategies and capacity building accordingly.

### Strengths and Limitations

This is a descriptive study in five mental health centres in different Eastern and Central Europe countries, hence the generalizability of the findings is uncertain. Moreover, all healthcare providers of all five CMHT volunteered to be part of a mobile team, so their personal commitment and their own motivation might influence the results positively compared to healthcare provider who are not involved in such a project. In addition, results may be driven by particular countries due to the difference in sample size. Due to relatively small sample sizes per country, country-specific analysis cannot be performed. Another limitation is that results are based on quantitative data only which do not really capture the scale of practice-level changes and the nuances entailed in it. Qualitative data, such as interview data, are needed to verify the findings. Data was collected after the health professionals and the peer workers received the first week of two-week training session. One week of training about the recovery approach may be unlikely to be sufficient to establish any real change in knowledge, attitudes, and appropriate actions. However, the findings reported in this manuscript focus only on the first step in the project plan, leading to the project aim. In fact, rather than relying on just one-week of training, the project entails additional ongoing support and supervision from the project partners which long term experience in implementing this model of care [36].

## Conclusion

After a one-week training session on community-based practices and collaborative teamwork, health workers had implemented key aspects of recovery-oriented practice, allocated roles and responsibilities. Training on its own can usually not sustain motivation and practice, so a highly motivated team is needed in order to embed newly learned skills into practice which was the case in this study. However, the role of nurses and peer workers needs to specify in more detail to improve their confidence level.

## Supplementary Information

**Additional file 1.**

## Data Availability

The dataset generated and analysed during the current study will not be made publicly available due to European Data Protection Law but maybe available by the corresponding author on reasonable request.

## Abbreviations

WHO: World Health Organisation
SMI: Severe Mental Illness
CMHT: Community Mental Health Team
RECOVER-E: Large-scale implementation of community-based mental health care for people with severe and enduring mental ill health in Europe
RSA-P: Recovery self-assessment tool Provider Version
TMSA: Team Member Self-Assessment Tool
