# Supplementary material for: Experiences of healthcare staff providing community-based mental healthcare as a multidisciplinary community mental health team in Central and Eastern Europe findings from the RECOVER-E project: an observational intervention study
Source: BMC Psychiatry. 2021 Oct 24;21:525. doi: 10.1186/s12888-021-03542-2 (PMC8543797; doi:10.1186/s12888-021-03542-2)
Supplement: Supplementary file 1 — Additional file 1. [file 12888_2021_3542_MOESM1_ESM.docx]

Supplementary Material

Characteristics of the study settings/project sites

|  | **Zagreb, Croatia** | **Kotor, Montenegro** | **Siret, Romania** | **Skopje, Macedonia** | **Sofia, Bulgaria** |
| --- | --- | --- | --- | --- | --- |
| **Catchment area (inhabitants)** | 980.000 | 89.000 (initially/at beginning of the study) 165.000 enlarged from October 2019** | 100.000 | 500.000 | 1.300.000**** |
| **Total number**  **of psychiatric**  **patients in**  **catchment area** | 12.226 | 9.000 (initially / at the beginning of thy study)  16.000 *enlarged / from October 2019*** | 51.000 | 30.000* | 27.000***** |
| **CMHT staff structure** | Psychiatrists, Psychologist, Nurses, Social Worker,  Non-mental health Professionals | Psychiatrists, Psychologist, Nurses, Social Worker | Psychiatrists, Psychologist, Nurses, Social Worker, Occupational therapists | Psychiatrists, Psychologist, Nurses, Social Worker, Psychiatric Trainees, Occupational Therapists, Disability Therapists | Psychiatrists, Psychologist, Nurses, Social Worker |
| **Type of services for people with mental health issues** | Inpatient treatment (acute and chronic care) Psychotherapeutic inpatient care,  Outpatient visits 1 per months and daily hospital for first episode psychosis and schizophrenia  Mobile team since 2017 | Inpatient and outpatient treatment (acute and chronic care)  Community based mental health service since 2010 | Inpatient and outpatient treatment (acute and chronic care) | Inpatient and outpatient treatment (acute and chronic care) | Inpatient and outpatient treatment (acute and chronic care) |
| **Financing of mental health services** | Croatian Health Insurance Fund  Out-of-pocket costs are not significant | National Health Insurance Fund (Bismarck’s model of financing) Funds from donations | Romanian Health Insurance Fund through the DRG system | Macedonian Health Insurance Fund | State financing Municipality National Health Insurance Fund and Out-of-pocket |

* Clinic of Psychiatry, Psychiatric hospital Skopje and outpatients MH Services

** Census of Population in Montenegro 2011 / http://www.monstat.org/userfiles/file/popis2011/saopstenje/saopstenje(1).pdf

*** Data gathered from hospital and mental health centres in each municipality (free estimation / there is no published source)

**** <https://www.nsi.bg/en/content/6704/population-districts-municipalities-place-residence-and-sex>

*****Data form NCPHA database (not published)
